# A practical approach to illustrate the importance of the bodily energy and heat balances and of the associated regulatory loops to healthcare students

**DOI:** 10.1186/s12909-026-09954-6

**Published:** 2026-07-20

**Authors:** Anne Riemann, Alexander Nolze, Michael Kopf, Gerald Schwerdt, Michael Gekle, Virginie Dubourg

**Affiliations:** Julius Bernstein Institute of Physiology, Martin Luther University Halle-Wittenberg, Halle (Saale), Germany

**Keywords:** Experimental learning, Thermoregulation, Metabolic rate, Climate change, Obesity

## Abstract

**Background:**

Training medical students is a demanding task, and some contents remain difficult to teach and understand. This also applies to topics that require increasing attention. For instance, obesity rates keep rising, while almost each year turns out to be hotter than the last. Consequently, it is inevitable that current healthcare students will one day be confronted with these challenges. But the underlying physiological principles are concepts that prove difficult to understand. To remedy this situation and improve students’ awareness and understanding of impacted patients, we have developed an educational tool to illustrate the concepts of metabolic rate and thermoregulation.

**Methods:**

Following a ‘learning by doing’ approach, we designed practical courses covering these topics and comprising data production, collection and analysis. Students measure their heart rates, metabolism rates (indirect calorimetry) and body temperature, at rest and during moderate-intensity exercise. They then (i) quantify the amounts of energy expended as muscular work or heat, (ii) characterize heat dissipation mechanisms, and (iii) estimate theoretical core temperature rise in the absence of thermoregulatory control. Their data are ultimately integrated into a large cohort dataset (*N* = 89), enabling the study of confounding variables (e.g. sex, body size). This course is evaluated by the students via an anonymized web-based survey.

**Results:**

Exercise-induced changes in heart rate showed that the test subjects were primarily healthy and challenged at a moderate level only. The measurements and subsequent calculations illustrated that this moderate-intensity exercise induced a strong increase in metabolic rate, and that this additional energy is spent as heat rather than muscle work. A dramatic shift in the relative contributions of the heat dissipation pathways during physical activity were also observed. Finally, average increase rates of core temperature of 1.33 °C/h at rest and 4.4 °C/h during moderate-intensity exercise were estimated. Only minor differences between female and male subjects were observed. This course has received very positive evaluations in recent years.

**Conclusions:**

We present a readily implementable approach that integrates education and science. It is designed to favor medical students’ comprehension of metabolic balance and thermoregulation while fostering critical evaluation of data quality and analytical rigor.

**Supplementary Information:**

The online version contains supplementary material available at 10.1186/s12909-026-09954-6.

## Background

 Energy is required for the basic functioning of the body and the maintenance of its structure (e.g. cardiac activity, respiration, resorption and secretion processes, digestion), for growth, for external work (motor activity), as well as for heat generation to maintain body temperature (thermoregulation). The body’s energy equilibrium is reached when energy intake equals energy needs. If energy intake exceeds energy expenditure, this is referred to as a positive energy balance, what is physiological during growth but can become pathological later on and lead to weight gain or even obesity. Current available data actually show that 16% of the adult world population was obese in 2022 and suggest an even stronger prevalence in the future if this epidemic is not slowed down [1]. This means that students currently being trained for professions in health care will face a continuously growing number of obese patients and deal with obesity-associated health risks. In order to take appropriate measures when consulting or treating such patients, future practitioners must understand how the human body uses energy, the concept of metabolic rate and how a disrupted energy balance associated with weight gain can affect bodily functions.

Furthermore, other challenges lie ahead of them, including caring for patients in an increasingly unstable climate. This latter has a negative influence on human health and wellbeing [2]. But climate change also directly influences the body’s energy balance. After all, the body releases most of the energy it converts as heat, which must then be dissipated in order to maintain body temperature. Extreme environmental conditions compromise the effectiveness of this aspect of the thermoregulation process, what can ultimately lead to serious health issues or even to death. The development of physiology-based models to adopt appropriate and quick safety measures and to reduce avoidable heat-related death has already been proposed, with a concept of focusing more on “hot people rather than hot weather” [3]. But to do so, a deep understanding of physiological concepts is required, hence the importance of appropriate trainings.

It is noteworthy that these challenges that await future practitioners are not actually unrelated. Indeed, a significant number of reports indicates a connection between global warming and obesity [4–8]. It is estimated that a 1 °C increase of annual mean temperature would lead to an increase in obese adults worldwide of 12% [4] due to a decreased physical activity and reduced adaptive thermogenesis, but also due to an impairment of food access [8, 9]. Therefore, the term of “syndemic”, a synergy of the obesity- and climate change-associated epidemics, has been proposed [8]. This is particularly alarming given the ageing of societies and the fact that older people are susceptible to overweight and to thermoregulation disorders, which are due to a reduced sweating capacity and a weakened cardiovascular system. Today’s obese young generation will be tomorrow’s elderly population, who will therefore suffer from multiple severe problems.

Given these imminent and apparently unavoidable challenges, it is essential that tomorrow’s health care staff understands the concepts of energy balance and thermoregulation, as well as the associated disruptions that can affect bodily functions.

These concepts are generally taught in physiology, where the curriculum includes water balance, body temperature regulation, and energy balance regulation. However, despite being critical for body homeostasis, these notions are pure integrative quantitative physiology and often remain abstract to students and even hard to teach [10]. To tackle this issue, we have applied the principle of “learning by doing”, which has been proven to have a positive impact on performance and on transfer of knowledge [11, 12], and developed a didactic tool in the form of practical classes. This approach allows students to test these concepts themselves and therefore to better understand them. These practical classes run in association with a series of corresponding lectures, which provides the students the required theoretical background, as well as seminars in which, for example, the physiology of older people is compared with that of young adults, such as our students, in order to put the illustrative results they have obtained during their practical sessions into context.

Additionally, by applying such evidence-based teaching we have the opportunity here to introduce students to larger sets of data and to raise awareness among them regarding data quality and proper data generation and interpretation. Doing so, we aim to train them to be able to identify reliable data and discriminate “real” differences (statistically significant) from differences by chances, what is critical for future medical staff that will be regularly confronted to the interpretation of clinical studies.

The energy and thermal balance-related experiments that are run described herein were designed with the following objectives in mind:

1. To measure metabolic rate and changes caused by moderate exercise.
2. To show the importance of heat transfer processes.
3. To show the influence of body size and sex on these parameters.
4. To prepare students to interpret and validate data.

We present here data collected over two successive university years, and discuss the different notions that our approach allows to pass on to medical students.

## Methods

### Participants and setting

Participants are students enrolled in the pre-clinical phase of the medical education program of the Martin Luther University Halle-Wittenberg, Germany. This program includes physiology practical classes taught to about 15 groups per year (with up to 20 students per group), that comprise a 60-min introductory seminar, 3 h of experimental work and 60-min of discussion of the results and integration of those in physiological and pathological contexts. The practical class including the exercise described here runs in conjunction with lectures and seminars about energy balance, thermoregulation and exercise physiology. In these classes, the physiology of older people and newborns is compared with that of young adults, such as our students. This helps to put into perspective and to generalize the type of measurements the students carry out during this practical course.

**Fig. 1:**
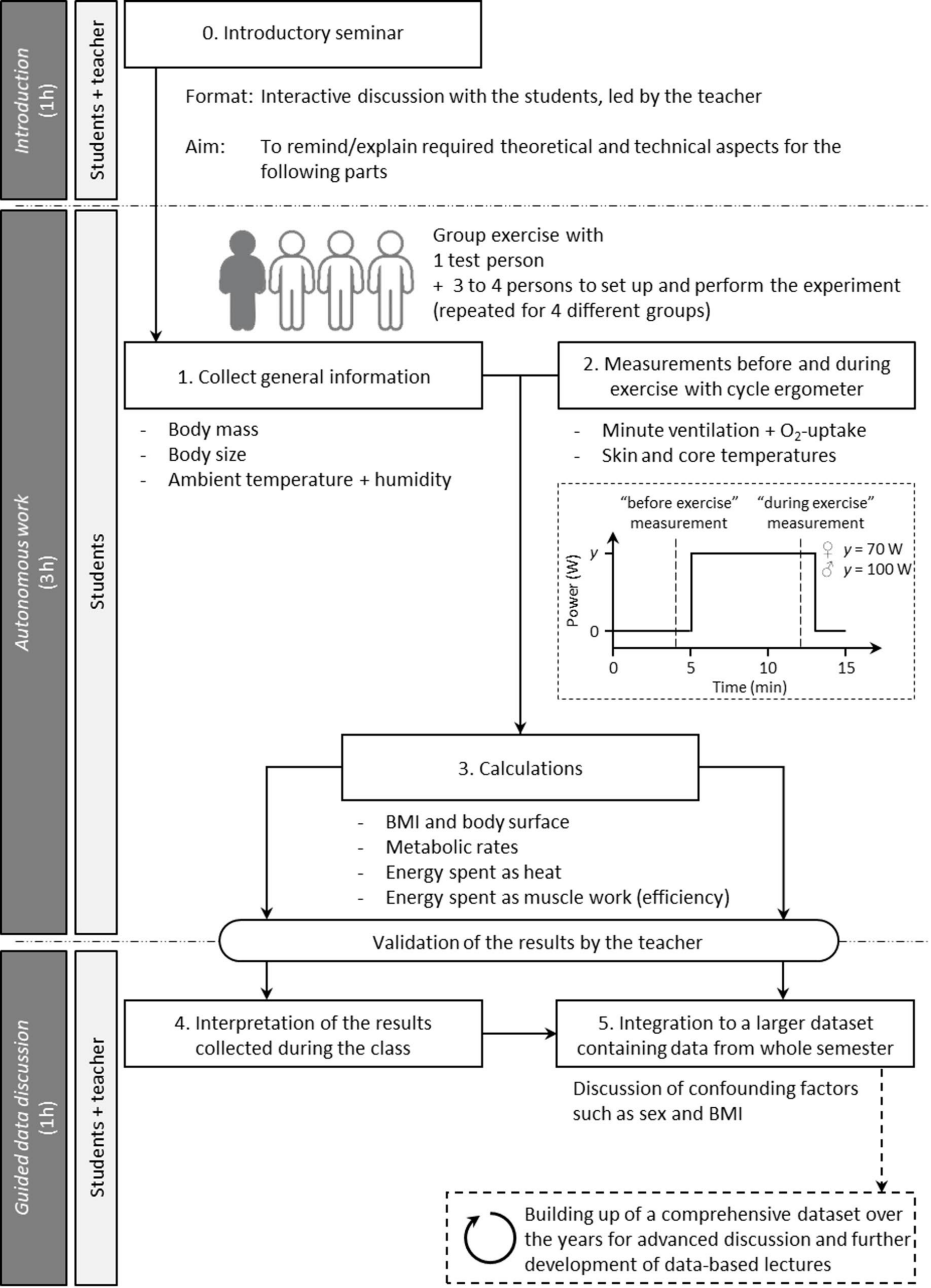
Flowchart describing the different phases of the practical class

Students form groups of 4 to 5 persons and carry out the experiment following the procedure illustrated in Fig. 1. Although participating to practical classes is compulsory, the role as test subject is voluntary. This experiment is performed up to four times per class. The data are collected anonymously (see also sample student worksheet in Supplementary Material). The Ethics Committee of the Medical Faculty-Martin Luther University Halle-Wittenberg (2025–203) approved the study.

It should be noted that, for safety reasons, we ask students with known medical conditions, such as asthma or cardiac arrhythmia, to refrain from taking part in experiments. More generally, students must attend a compulsory safety seminar at the start of the physiology curriculum, during which they are advised, amongst other things, on how to proceed if they believe their state of health could pose a problem whilst acting as a test subject. Students have the opportunity to discuss any concerns regarding potential subclinical conditions with their lecturers before taking part in an experiment as a test subject. That said, students with such conditions are guaranteed that they will not face any form of discrimination or exclusion within the group.

### Infrastructures and required material

The practical class comprising the method described here takes place in a classroom equipped with a cycle ergometer and the necessary equipment to assess minute ventilation, oxygen (O_2_) consumption and body temperature (Table 1).

**Table 1:** Required material for data acquisition and collection

Required material	References
Infrared ear thermometer	Bosotherm medical, BOSCH + SOHN GmbH u. Co. KG, Jungingen, Germany
Thermo-/Hydrometer	Eurochron ETH 5500, Conrad Electronics, Hirschau, Germany
Heart rate sensor	Polar H7 (Polar Electro Oy, Kempele, Finland)
Cycling ergometer	ER900 (ergoline GmbH, Bitz, Germany)
Breathing mask for Spiroergometry	CORTEX Breathing Masks Size M, L (Cortex Biophysik GmbH, Leipzig, Germany)
Spiroergometry system with breath-by-breath technology	Cortex Metalyzer 3B R3 (Cortex Biophysik GmbH, Leipzig, Germany)
Spiroergometry-associated software	MetaSoft Studio software (Cortex Biophysik GmbH, Leipzig, Germany)
Computer (to acquire and collect data)	--

### Procedures

Before joining the class, the students receive a protocol, a table with the list of data to collect and the equations required to calculate some data-derived parameters (example provided in Supplementary Material – translated from German).

#### Procedure part 1: collect general information

A measuring rod and a scale are available for the students to measure their body height and weight during the class. A thermometer and a hygrometer are set in the classroom for them to know the ambient temperature (T_room_) and humidity.

#### Procedure part 2: measurement before and during exercise with cycle ergometer

An infra-red thermometer is used to measure the core temperature (in auditory canal - T_core_) and the skin temperature (on the forearm - T_skin_) of the test persons.

A person’s metabolism rate can be determined using indirect calorimetry. Indeed, the usable energy (ATP) is essentially provided by oxidative phosphorylation, i.e. by oxygen consumption. The oxygen intake (= inhaled O_2_ - exhaled O_2_) is therefore almost equal to the amount of oxygen consumed by oxidative phosphorylation of fat, carbohydrate, proteins. It is thus used as a measure of the energy metabolism and reacts immediately to a change in energy requirements. Breath-by-breath cardiopulmonary and heart rate data were collected.

A cycling ergometer was used to apply the exercise protocol. The 18 min long protocol started with a 5 min resting phase, followed by an 8 min exercise period with a cycle power of 70 W for women and 100 W for men, and finally a 5 min resting phase.

#### Procedure part 3: calculations

The following paragraphs describe the equations used to estimate various variables, including the amount of heat lost through the skin’s surface or via respiration. These equations are based on current knowledge in physiology and on the equations described, for example, by Jessen et al. [13] That said, in order to make these complex calculations accessible to early-stage students, we have adapted some of these equations using fixed coefficients (e.g. body surface involved in a given type of heat loss). The values obtained are therefore not precise physical values but realistic approximations from a physiological point of view, which are used for educational purposes.

### Body parameters

The BMI and body surface area (BSA, according to the method Du Bois and Du Bois [14]) of the test persons are calculated as shown in Eqs. (1) and (2).

1 $$\mathrm{BMI}=\mathrm{body}\:\mathrm{weight}\:\left[\mathrm{kg}\right]/\mathrm{body}\:\mathrm{height}^{2}\left[\mathrm{m}^{2}\right]$$

2 $$\begin{aligned}\mathrm{BSA}&=\mathrm{body}\:\mathrm{weight}^{0.425}\:\left[\mathrm{kg}\right]\\&\times\mathrm{body}\:\mathrm{height}^{0.725}\left[\mathrm{cm}\right]\times0.007184\end{aligned}$$

### Metabolic rate determination

The metabolic rates (MR - before and during exercise) of the test persons are determined based on their O_2_-uptake, according to the principle of indirect calorimetry (Eq. 3).

3 $$\begin{aligned}\mathrm{MR}\:\left[\mathrm{J/s}\right]&=\mathrm{O}_{2}-\mathrm{uptake}\left[\mathrm{L/min}\right]\\&\times\text{caloric equivalent}\left[\mathrm{kJ/L}\left(\mathrm{O}_{2}\right)\right]\\&\times1000/60=\mathrm{O}_{2}-\mathrm{uptake}\left[\mathrm{L/min}\right]\\&\times20\times1000/60\end{aligned}$$

Given the conditions under which the measurements described here are carried out (during practical sessions), it is only possible to measure rest and exercise metabolic rates. The basal metabolic rate measurements require specific conditions (e.g. thermal neutrality, rest, no exertion, fasting) that cannot be achieve here. We nevertheless propose an estimate of it that we name “Non-heat- or breathing-related metabolic rate” (see description below).

### Body surface-associated heat loss

The quantities of energy spent through body surface-related heat loss is calculated using the Eq. (4, 5, 6, 7 and 8), which were adapted from Jessen [13]. The latter provides specific coefficients for each type of heat loss that represents the heat transfer between components of a given system or, in simple terms, reflect the ‘efficiency’ of that mechanism in dispersing heat. Additionally, different factors are used to correct the body surface area available for each type of heat loss.

Radiative heat loss rate [J/s] = radiative heat-transfer coefficient [J/(s × °C × m²)] × temperature difference [°C] × body surface available for radiative heat exchange [m²].

4 $$\text{Radiative heat loss }[\mathrm{J/s}]=6\times\left(\mathrm{T}_{\mathrm{skin}}-\mathrm{T}_{\mathrm{room}}\right)\times\left(\mathrm{BSA}\times0.5\right)$$

Convective heat loss rate [J/s] = convective heat-transfer coefficient [J/(s × °C × m²)] × temperature difference [°C] × body surface available for convective heat exchange [m²].

5 $$\text{Convectiveheat loss }[\mathrm{J/s}]=2.5\left(\mathrm{T}_{\mathrm{skin}}-\mathrm{T}_{\mathrm{room}}\right)\times\left(\mathrm{BSA}\times0.3\right)$$

Evaporative heat loss rate [J/s] = evaporative heat-transfer coefficient [J/(s × °C × m²)] × H_2_O-vapor pressure difference [mm Hg] × body surface available for evaporative heat exchange [m²].

6 $$\text{Evaporheat loss }[\mathrm{J/s}]=40\times\left(\mathrm{P}_{\mathrm{skin}}-\mathrm{P}_{\mathrm{room}}\right)\times\left(\mathrm{BSA}\times{f}\right)$$

with *f* the estimated fraction of skin covered by sweat and contributing to evaporative heat loss. It is a condition-dependent factor equal to 0.3 for the measurement before the exercise (our practical classes take place in summer, with room temperatures still comprised between 22 and 28 °C, suggesting a minimal basal sweating rate with ≤ 30% of wet skin [15]) and 0.9 during the exercise (sweating increases visibly with physical work, meaning that the fraction of skin involved in the evaporation process increases [16]). Based on the Magnus formula, the actual H_2_O-vapor pressure on the skin (P_skin_) and in the ambient air (P_room_) is calculated as follow:

7 $$\begin{aligned}\mathrm{P}\:\left[\mathrm{kPa}\right]&=0.6111213\times\mathrm{e}^{17.5043{\times\mathrm{T}\left[^\circ\mathrm{C}\right]}/\left(241.2+\mathrm{T}\left[^\circ\mathrm{C}\right]\right)}\\&\times\mathrm{humidity}\left[\%\right]/100\end{aligned}$$

Before the exercise, the relative humidity of the skin is considered as equal to the one in the air. During the exercise, it is set at 100%.

The total amount of energy dissipated as heat via the body surface is also calculated by adding together the hereinabove values.

8 $$\begin{aligned}&\text{Total body surface-associated heat loss}\left[\mathrm{J/s}\right]\\&=\text{Radiative heat loss rate}\left[\mathrm{J/s}\right]\\&+\text{Convective heat loss rate}\left[\mathrm{J/s}\right]\\&+\text{Evaporative heat loss rate}\left[\mathrm{J/s}\right]\end{aligned}$$

### Respiration-related energy expenditure

The amount of heat lost by respiration is estimated based on the measured minute ventilations of the test persons (Eq. (9)). The H_2_O-vapor in air is derived from the ideal gas law (H_2_O-vapor = (PV)/(RT) x water molar mass, with P derived from Magnus formula – see (7) –, V = 1 L and water molar mass = 18.02 g). As an example, the inhaled air in a room at 25 °C with 60% humidity contains 14 mg water /L air, while the exhaled air contains 44 mg/L (air at 37 °C, 100% humidity) [17]. The difference is thus of about 30 mg/L. We calculated this value, taking into account various conditions met in practical class and, set it as a constant value equal 25 mg/L (corresponds to the minimal value calculated).

Respiratory heat loss [J/s] = (Minute ventilation [L/min] / 60) × (H_2_O-vapor in exhaled air - H_2_O-vapor in inhaled air [mg(H_2_O)/L(air)]) × heat of water vaporization [kJ/g(H_2_O)]

9 $$\text{Respiratoraheat loss }[\mathrm{J/s}]=\left(\text{Minute ventilation}\left[\mathrm{L/min}\right]/60\right)\times25\times2.5$$

### Non-heat- or breathing-related metabolic rate

We defined the amount of energy used by the test persons for posture and other basic functions of their body (including here also e.g. digestion) as the “non-heat or breathing-related metabolic rate” (NHB-MR). This is estimated by calculating the differences between the metabolic rate and the amounts of energy spent as heat (Eq. 10).

10 $$\begin{aligned}\mathrm{NHB-MR}\left[\mathrm{J/s}\right]&=\mathrm{MR}\left[\mathrm{J/s}\right]-\text{total body}\:\\&\mathrm{surface-associated}\text{heat loss}\left[\mathrm{J/s}\right]\\&-\text{respiratory heat loss}\left[\mathrm{J/s}\right]\end{aligned}$$

For the measurements during exercise, the power set in the cycle ergometer is also subtracted (70–100 W for female and male test subjects, respectively).

### Net efficiency

The net efficiency is defined as the relative amount of energy actually spent as muscle work during the exercise phase (Eq. 11).

11 $$\begin{aligned}\text{Net efficiency}\left(\%\right)&=100\times\text{power in ergometer}\\&\left[\mathrm{J/s}\right]/\left(\mathrm{MR}_{\mathrm{during}}\left[\mathrm{J/s}\right]-\mathrm{MR}_{\mathrm{before}}\right)\end{aligned}$$

### Theoretical core temperature increase in case of failing body surface-related heat transfer

If heat dissipation via the skin (by radiation, convection and evaporation) or breathing were fully impaired, the core temperature would increase over time. This theoretical increase is estimated for the test subjects in the two measurement phases using the Eq. (12) (adapted from Boron and Boulpaep [18]).

Core temperature increase [°C/h] = 3.6 x total heat loss [J/s] / (body heat capacity [kJ/(kg x °C)] x body weight [kg])

12 $$\begin{aligned}\text{Core temperature increase }[\mathrm{C}^{\circ}/\mathrm{h}]=\\3.6\times\text{total heat loss}\left[\mathrm{J/s}\right]/\left(3.5\times\text{body weight}\left[\mathrm{kg}\right]\right)\end{aligned}$$

### Sweat production rate

The sweat production is estimated as described in Eq (13).

Sweat production rate [mL/h] = Evaporative heat loss rate [J/s] x 3600 / heat of evaporation of sweat [19] [J/mL].

13 $$\begin{aligned}\text{Sweat production rate }[\mathrm{mL/h}]=\\\text{Evaporative heat loss rate}\left[\mathrm{J/s}\right]\times{3600}/2500\end{aligned}$$

### Data analysis and interpretation

Initially, only the results of the test persons obtained during their practical class were shown to the complete group and discussed (gathered in a table file, see Supplementary Material for example). But the data variability and the identification of confounding factors (e.g. sex, body size of the test subjects) supported the idea of collecting data and building overtime a larger dataset in order to discuss more global patterns and exercise-induced changes with the students.

However, before integrating data from a practical class into this larger dataset, the instructor of the days runs a plausibility-check. They exclude outlier values (i.e. physiologically senseless or technically unsound) that cannot be corrected by re-analyzing the indirect calorimetry raw data (e.g. by making sure of selecting values in ranges without any artefacts) and that most probably result from bad data collection. The data of three different test persons were excluded based on this principle (e.g. core temperature below 35 °C, total body surface-associated heat loss values higher that the corresponding metabolic rates, duplication of the results of a test person). This means that out of the data initially collected for 92 test persons (over two university semesters), 89 of them were included. The data corresponding to these 89 test persons were included throughout the different analysis steps (no missing values) and are included in the supplementary material.

The statistical analysis for the complete dataset were performed with Rstudio (R version 4.4.2). Paired and unpaired analyses were implemented if the compared values belonged to one individual (before and during exercise) or to different individuals (e.g. men vs. women), respectively. Wilcoxon signed rank test was used for paired comparisons and Wilcoxon rank sum test for two-group comparisons. The exact (computed) p-values are displayed. No multiple correction was applied. Additionally, the correlation of different parameters was assessed using the Spearman method. This method was chosen over Pearson’s method because not all compared traits were normally distributed (tested with Shapiro-Wilk normality test). Cohen’s d was used as a measure of effect sizes.

### Evaluation

The reception by students and the benefits of the method described here are evaluated in two different ways.

1. At the end of the class itself, the students must pass a test made of four multiple-choice questions related to the topic (example provided in Supplementary Material – translated from German). The proportion of students who passed the examination is an indicator of their understanding of the topics covered.
2. A web-based evaluation takes place at the end of the semester. Students can give it marks comprise between 1 (very good) and 5 (very poor) for (a) the course overall and (b) its learning effect. This assessment is voluntary and anonymous to ensure its objectivity.

## Results

### Description of the cohort group

Data presented here were collected for 89 test persons (including 31 men and 58 women) over two successive university years (2024 and 2025). This subgroup is representative of a larger and more global student population of the medical faculty of the Martin-Luther University Halle-Wittenberg, whose data were collected among our students over 11 years (comprises 2061 subjects). This is the case for the sex distributions, the average body mass indexes (BMI) and ages (Table 2).

**Table 2:** The participants whose results are presented here are representative to the ensemble of our students

		Sex		BMI (kg/m²)		Age (years)	
	Number of participants	Men (%)	Women (%)	Mean	95% CI	Mean	95% CI
Total	2061	36	64	22.24	22.11–22.38	22	21–22
Subgroup	89	35	65	22.19	21.52–22.86	22	21–22

The heart rates of the test subjects were measured throughout the procedure. The obtained values were used to estimate the exercise burden applied on them and their health status. The heart rates of all students displayed a reliable increase when exercise started and reached a stable steady state during the exercise phase. These results indicate that the intensity of the physical exercise was not overly strenuous for any of them. According to the recommendations of the American Heart Association (https://www.heart.org/en/healthy-living/fitness/fitness-basics/target-heart-rates - accessed in September 2025), the heart rate during moderate-intensity physical activities should be about 50–70% of maximum heart rate (maximum heart rate ≈ 220 – age of the test subject). The values obtained for the student cohort therefore confirmed that the exercise to which they were subjected was of moderate intensity only (Table 3). Additionally, the recovery of the heart rate immediately after exercise (here after 60 s - HRR60 = HR_end of exercise_ – HR_end of exercise + 1 min_) has been defined as a fitness-parameter and values below 12 bpm associated with increased mortality and cardiovascular-associated pathologies [20, 21]. The values obtained confirm that the subjects are primarily healthy (Table 3).

**Table 3:** Heart rate-derived values of the participants. The heart rates reached by the students during the exercise phase indicate that the physical activity to which they were subjects was of moderate intensity only (% maximum HR). Heart rate recovery 60 (HRR60) scores suggest that all participants were primarily healthy. (Number of participants *N* = 89)

		Mean	95% CI
	HR at rest	83	81–86
	HR during exercise	131	128–135
Indicator of physical load	% maximum HR	66	65–68
Indicator of fitness	HRR60	28	26–30

### Metabolic and thermal response to moderate-intensity exercise

**Fig. 2:**
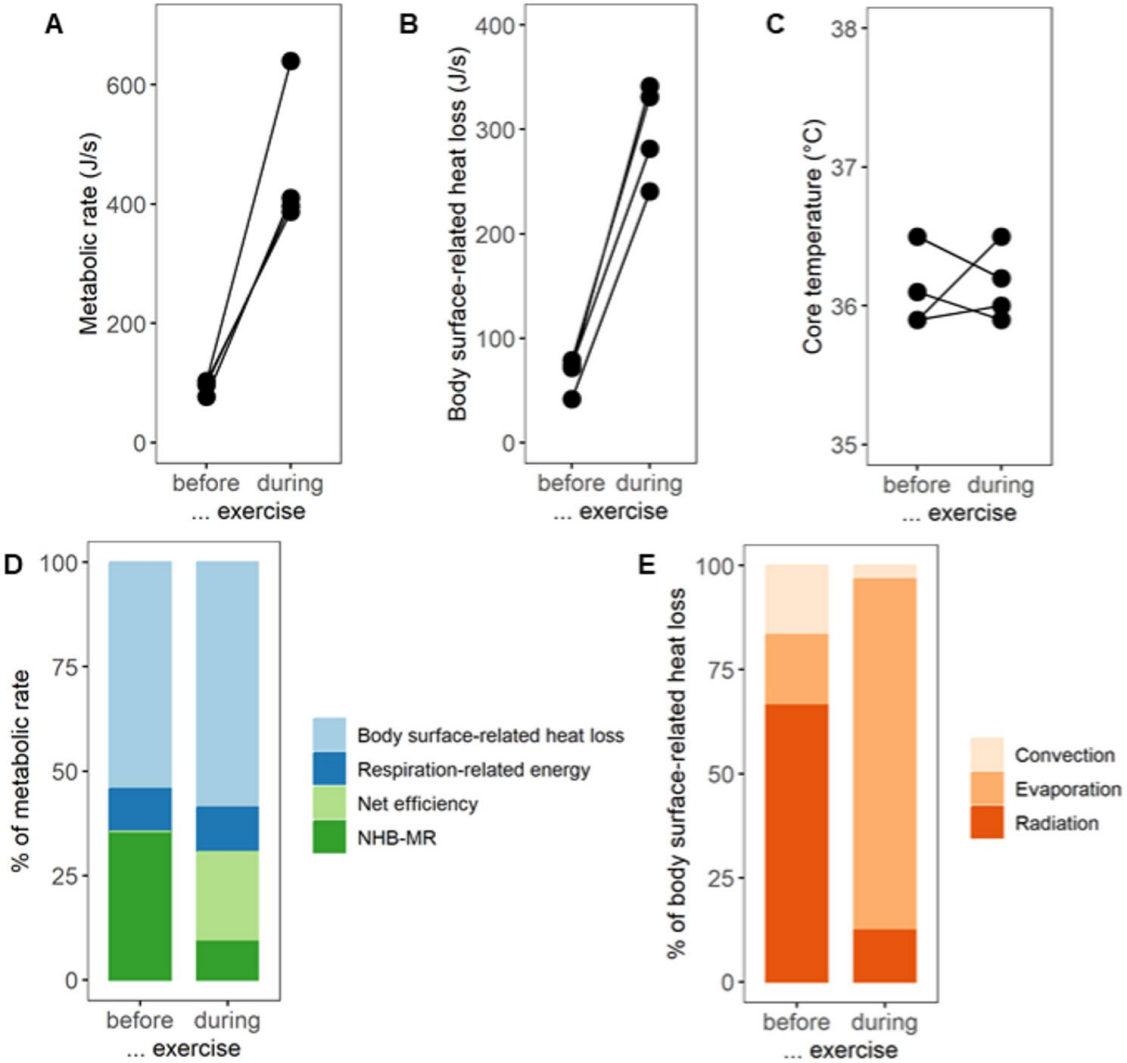
Results obtained during one single practical class. The figures display the exercise-induced changes in (**A**) metabolic rate, (**B**) heat loss at the body surface and (**C**) core temperature. (Number of test persons *N* = 4) (**D**) The results of one of the test persons are discussed to highlight how energy is spent at “rest” and during the exercise. The relative amounts of energy lost by heat transfer or while breathing are calculated. The muscle work required to complete the physical activity is defined as “net efficiency”. The rest is defined as “Non-heat- or breathing-related metabolic rate” (NHB-MR). **E** The relative efficiency of each of heat dispersion mechanism is estimated and compared for the “resting” and exercise phases

During the practical class, the students have the task to calculate body parameters (e.g. BMI and body surface) of their test subject, but also to determine the metabolic rate and the energy expenditure of this person, both at “rest” (before the exercise, while the person sits calmly on the cycle ergometer) and during exercise (Fig. 1). Figure 2 shows results typically obtained during a single practical class, during which the experiment is repeated for four test persons.

When discussing these results, the students can already observe that the metabolic rate of all test subjects and therefore their amount of spent energy increases considerably from physical activity, despite the moderate intensity of the latter (Fig. 2A). The results also show that this strong increase in metabolic rate comes along with an increase in heat dissipation at the body surface (Fig. 2B), while the core temperature remains unchanged if considering all test subjects (Fig. 2C).

In a second discussion phase, example values of one of the four test subjects are usually used to illustrate fundamental points. First, we show that the relative amounts of energy spent as heat (at the body surface or by breathing) are actually similar in the calm/resting phase before the exercise as during the physical activity (Fig. 2D). Conversely, the amount of energy spent as actual muscle work during the cycle ergometer exercise (net efficiency) is, often to the surprise of the students, below 30%.

Finally, the way heat is dissipated at the body surface before and during physical activity is discussed (Fig. 2E). With these data, it can clearly be shown to students that, although the relative amount of energy lost as heat at the body surface is similar in both phases, the mechanisms through which it occurs drastically change.

However, the data collected during a single practical class do not allow discussing any statistically significant changes due to the reduced sample size and a high variability of the data, as illustrated by the measurement of the core temperature (Fig. 2B). The former aspect is discussed with the students in order to illustrate the need of gathering data from a sufficient number of participants before being able to draw conclusions. On the other hand, the variability of the core temperature measurement is typically used to demonstrate to the students the need to perform replications and to carefully interpret single-point measurements for a given person.

Meanwhile, we have observed that students often have difficulties evaluating data, especially when it comes to assessing the effect size and distinguishing between “real” changes compared to “noise” signal or “changes by luck”. A critical, structured and in-depth assessment of the data by the students is a vast enrichment of the course but needs an increase in sample size and a structured guidance from a teacher. Thus, in order to convey more advanced concepts to our students, the data are directly integrated into a larger dataset that contains data acquired in previous practical classes. The teacher is then free to choose which aspects they wish to develop with their group of students, selecting certain sections to bring into focus and addressing physiological patterns that his/her actual students find most difficult to understand.

### Influence of body parameters and sex on metabolic rate at “rest”

**Fig. 3:**
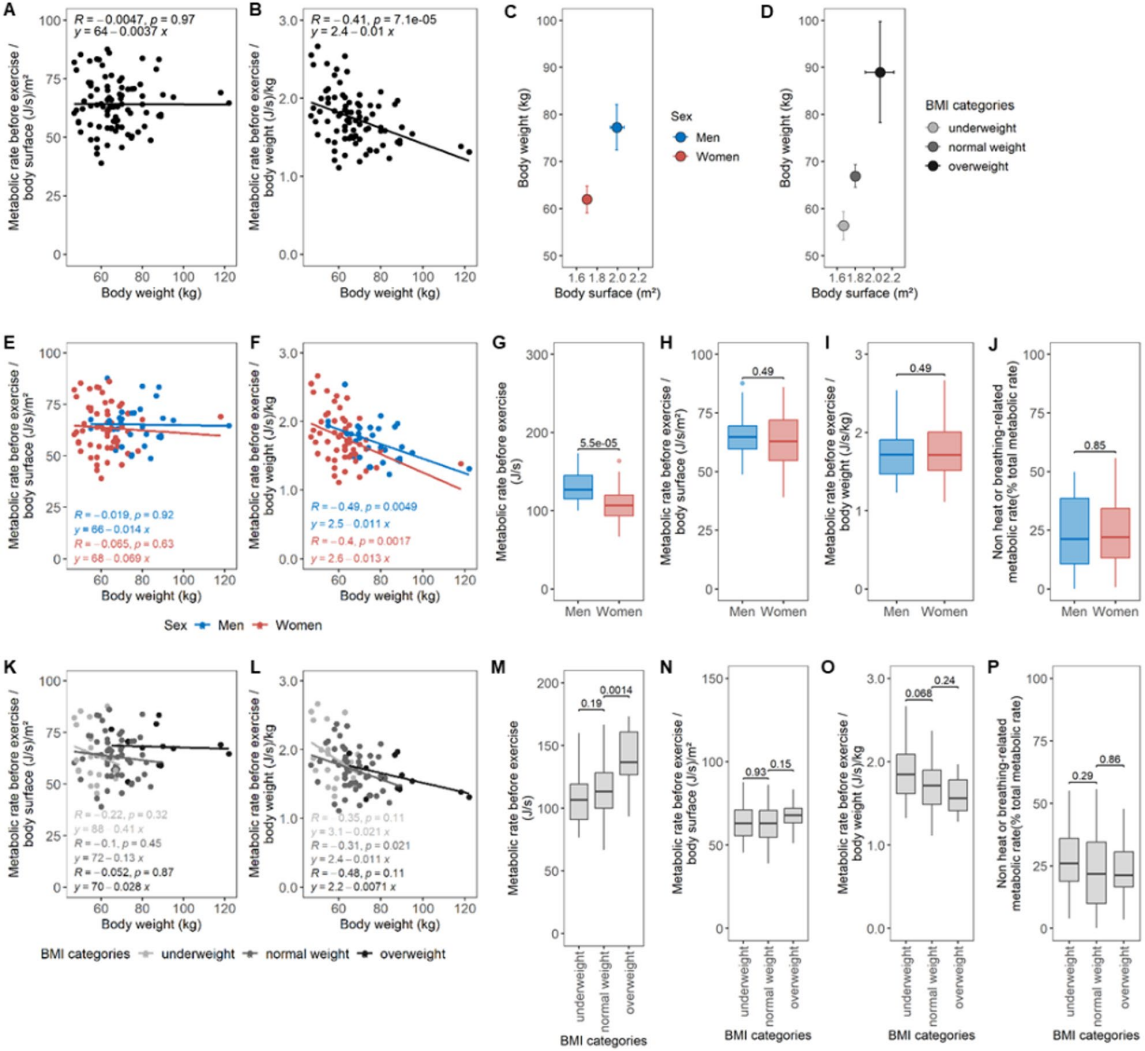
Global dataset and influence of body parameters and sex on metabolic rate at “rest”. **A-B **Correlation between body size (here as body weight) and metabolic rate corrected for (**A**) body surface or (**B**) body weight. **C** Average body weight and body surface of men and women (mean ± 95% CI). **D** Similar observations are made for individuals with different body types (defined according to their BMI). **E-F** Correlations between body size and metabolic rate corrected for (**E**) body surface or (**F**) body weight for each sex group. **G** Metabolic rate before exercise differed between men and women, due to sex-associated differences in (**H**) body surface and (**I**) body weight. **J** Relative NHB-MR of men and women before exercise. **K-L** Correlations between body size and corrected metabolic rates for the different BMI categories. **M** The “resting” metabolic rate of subjects in different BMI categories are compared to see if their resting metabolism depends on their body build (Underweight, BMI < 20, *n* = 22 – normal weight, 20 ≤ BMI ≤ 25, *n* = 55 – Overweight, BMI > 25, *n* = 12, includes 2 persons with BMI > 30 and theoretically categorized as obese). This comparison is repeated after correction for (**N**) the body surface or (**O**) the body weight of the individuals. P Relative NHB-MR of the different BMI categories. (Number of participants *N* = 89)

A possibility is to start by discussing the results obtained before the exercise phase, at “rest”. First, we show that after adjusting the metabolic rate according to body surface area, the values obtained are not influenced by the person’s size (Fig. 3A). On the contrary, if the metabolic rate is adjusted for the body weight, the values obtained remain a function of the person’s size: the heavier a person is, the lower the energy turnover per kg (Fig. 3B). In this “rest” phase, most of the produced energy is not used to maintain posture and vital functions, but is actually dissipated as heat in the surroundings with the ultimate goal to maintain the core temperature at a constant level (example in Fig. 2C). Instead of simply giving students the well-known example of newborn babies, whose low surface area to volume ratio leads to poor thermoregulation and rapid heat loss, the data we obtained from the participants directly demonstrates that the body surface area is indeed more decisive in the heat dissipation process at rest than the actual weight of the person. Moreover, this implies that to compare the heat dissipation efficiency of different individuals, a correction of the measured metabolic rate for the body surface area should be applied, while a correction for the body weight will provide more information regarding the energy turnover of the tissues themselves.

With this is mind, we can then discuss what happens if these data are stratified according to confounding factors, especially the sex and the body type of the test subjects since both correlate with different body size parameters (Fig. 3C-D). For instance, men and women divide into two groups with clear cuts regarding body weight and surface (Fig. 3C). Despite these differences, the corrected metabolic rates of men and women show the same correlation trends with body surface area and weight (Fig. 3E-F). The values obtained by the students during their calculations suggest that men have a higher resting metabolic rate than women, as often described in textbooks (Fig. 3G). However, this difference vanishes after correction for the body surface area (Fig. 3H): men have a higher resting metabolic rate, meaning that they produce more heat, but they globally have a larger body surface area to “evacuate” this heat-excess compared to women. The sex-associated differences also vanish after correction of the metabolic rate for body weight (Fig. 3I). This comes rather unexpectedly since we previously showed a dependency between the corrected metabolic rate and body weight (Fig. 3A-B) and therefore expect that the values for women, whose body weight is generally in the lower range (Fig. 3C), are higher than that of men. It nonetheless could be explained by the fact that BMI-matched men and women mostly have different tissue composition, with men having more lean mass and women more fat mass [22].

Using the values obtained when the students sit still and calm at the beginning of the experiment, we can also estimate the metabolic rate necessary for structure maintenance and bodily functions, but not involved in heat loss. We defined this value as the “non-heat or breathing related metabolic rate” (NHB-MR). It should nonetheless be noted that the practical classes take place directly after lunch and in summer, meaning that NHB-MR also include digestion and eventually thermoregulation, although the room temperature remained below 30 °C. It is therefore only a very rough estimate of the basal metabolic rate, which must be measured in standardized conditions. To calculate it, the amounts of energy spent through heat dispersion (at the body surface or in the lungs) are subtracted from the metabolic rate values measured at “rest”. The relative amount of energy associated with NHB-MR is similar for men and women (Fig. 3J).

The same parameters are also explored for persons with different body types, defined here according to their BMI categorization. The association between corrected metabolic rates and body weight did not differ between these categories (Figs. 3K-L). The metabolic rate of the persons in overweight is higher than that of persons with a so-called “normal” weight (Fig. 3M). Interestingly, there is no difference when comparing the reference group to the underweight one. This is due to a steeper increase of the body surface area from normal-to-overweight than from underweight-to-normal (Fig. 3D - median body surfaces equal 1.7, 1.8, 2.1 m² for underweight, normal weight and overweight groups, respectively). This assumption is confirmed when adjusting the metabolic rate for body surface, since no difference is visible anymore (they have more body surface area to dissipate the excess of heat generated by their higher metabolic rate) (Fig. 3N). On the other hand, metabolic rate correction for body weight shows a reverse trend (Fig. 3O), matching the association made previously (Fig. 3B). This nevertheless suggests that the more corpulent a person is, the lower the energy turnover per kilogram of body mass. This is adequate considering that overweight/obese individuals have in comparison to normal weight individuals more fat mass, which has a lower energy turnover than lean mass. The NHB-MR is nonetheless similar for all BMI categories (Fig. 3P).

To summarize concerning the discussion based on the dataset acquired in “resting” conditions by the students themselves, we have the possibility here to illustrate concretely that….

- Heat dissipation is a function of body surface area rather than body weight.
- The generally bigger body surface area of men compensates for a higher resting metabolic rate compared to women, by enabling a better heat dissipation.
- Similarly, the larger body surface area of overweight individuals compensates for a higher resting metabolic rate.
- The corrections of metabolic rate values for body weight provide hints concerning the relative amount of fat and lean mass of a certain group compared to another.

### Influence of body parameters and sex on exercise-induced changes in metabolic rate and heat loss

Alternatively, one can also use the dataset to discuss exercise-induced changes in metabolic rate and heat loss, and again the putative roles of sex and body type.

### Exercise-induced changes in metabolic rate

**Fig. 4:**
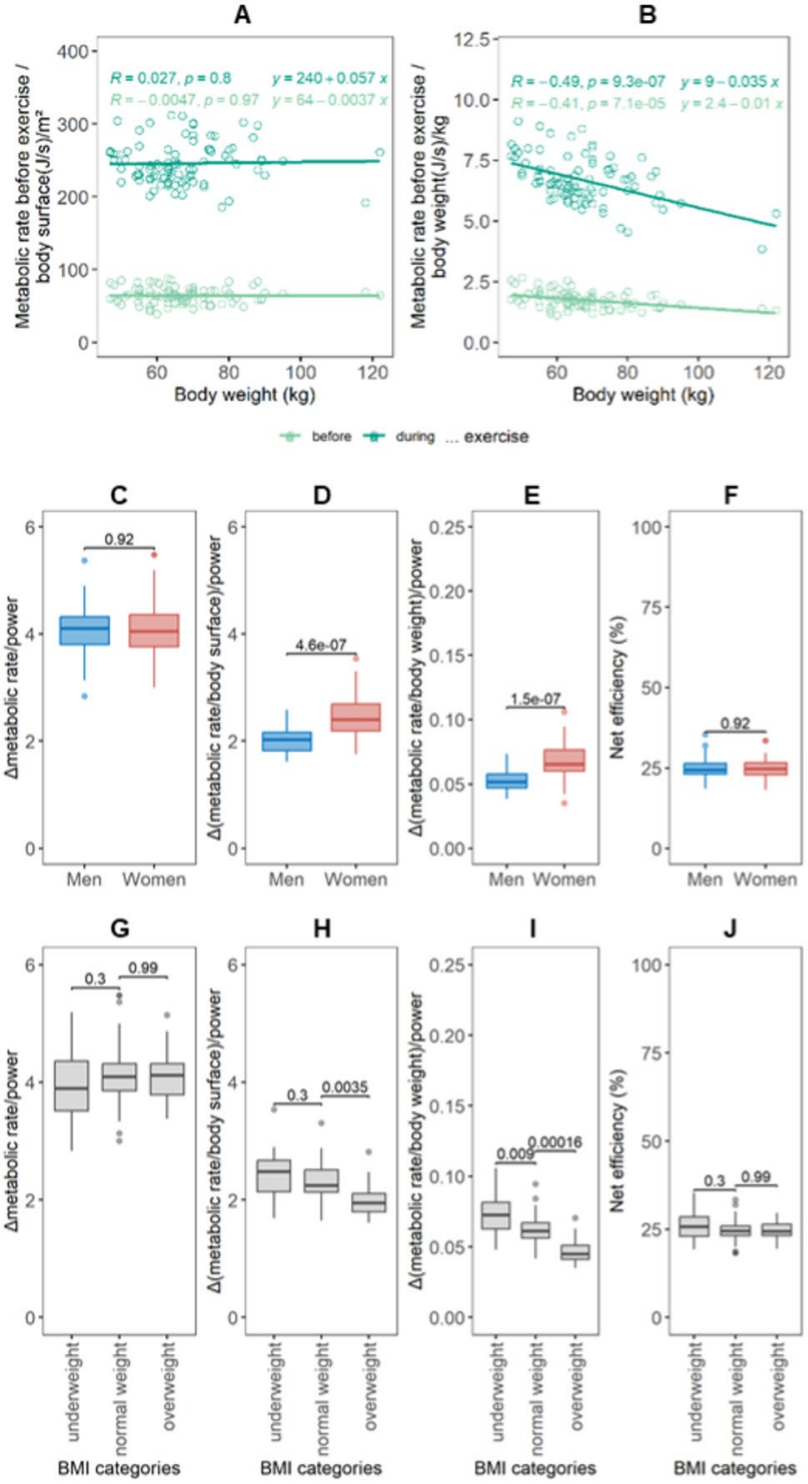
Exercise-induced changes in metabolic rates and the influence of body parameters and sex. **A-B** Correlation between body size (here as body weight) and metabolic rate corrected for (**A**) body surface or (**B**) body weight, before and during the exercise phase. **C** The increase of metabolic rate required to achieve the physical work (Δmetabolic rate / power) for men and women. These values were also normalized for (**D**) body surface or (**E**) body weight. **F** The relative amount of energy invested in the physical activity. **G** The increase of metabolic rate required to achieve the physical work (Δmetabolic rate / power) for the different BMI categories (Underweight, BMI < 20, *n* = 22 – normal weight, 20 ≤ BMI ≤ 25, *n* = 55 – Overweight, BMI > 25, *n* = 12, includes 2 persons with BMI > 30 and theoretically categorized as obese). These values were also normalized for (**H**) body surface or (**I**) body weight. **J** The relative amount of energy invested in the physical activity for the different BMI categories. (Number of participants *N* = 89)

First, we show that, despite the moderate-intensity of the exercise, the metabolic rates of the test subjects strongly increase (about 4-fold-increase in average) (Figs. 4A-B). We additionally observe that the body weight dependency is even more accentuated during the exercise than at “rest”.

Because men and women are not subject to the same exercise intensity (cycle ergometer set at 70 W and 100 W for women and men, respectively), the exercise-induced increase in metabolic rate (Δmetabolic rate) is corrected accordingly before comparing the data for both sex groups (Δmetabolic rate / power). Contrary to the students’ expectation, the results first show no difference between men and women (Fig. 4C). Even more unsettling for the students, when body surface area or body weight are also taken into account, the exercise-induced increase turns out to be greater in women (Figs. 4D-E). This suggest a difference in the energy turnover of men and women. On the other hand, what was observed for a single student (Fig. 2C) is confirmed with the larger cohort: the exercise-related pure muscle work corresponds only to 25% of the energy spent, and is the same for both men and women (Fig. 4F).

The exercise-induced changes in metabolic rate are also compared for subjects in different BMI categories (Figs. 4G-I). No difference is reported for power-corrected increases in metabolic rate (Δmetabolic rate / power), suggesting first that the metabolic rate increase results only from muscle work activation and that the body size of the individuals does not play a role. However, corrections for body surface area or body weight show a reduced effect for the overweight category. The lower metabolic rate increase per body surface area could possibly be explained by the fact that the larger the body surface of a person is, the more efficient is heat dissipation, and overweight individuals have an over-proportional body surface (Fig. 3D). The exercise-related pure muscle work is nonetheless similar throughout all categories and remains again about 25% (Fig. 4J).

### Exercise-induced changes in heat loss mechanisms

**Fig. 5:**
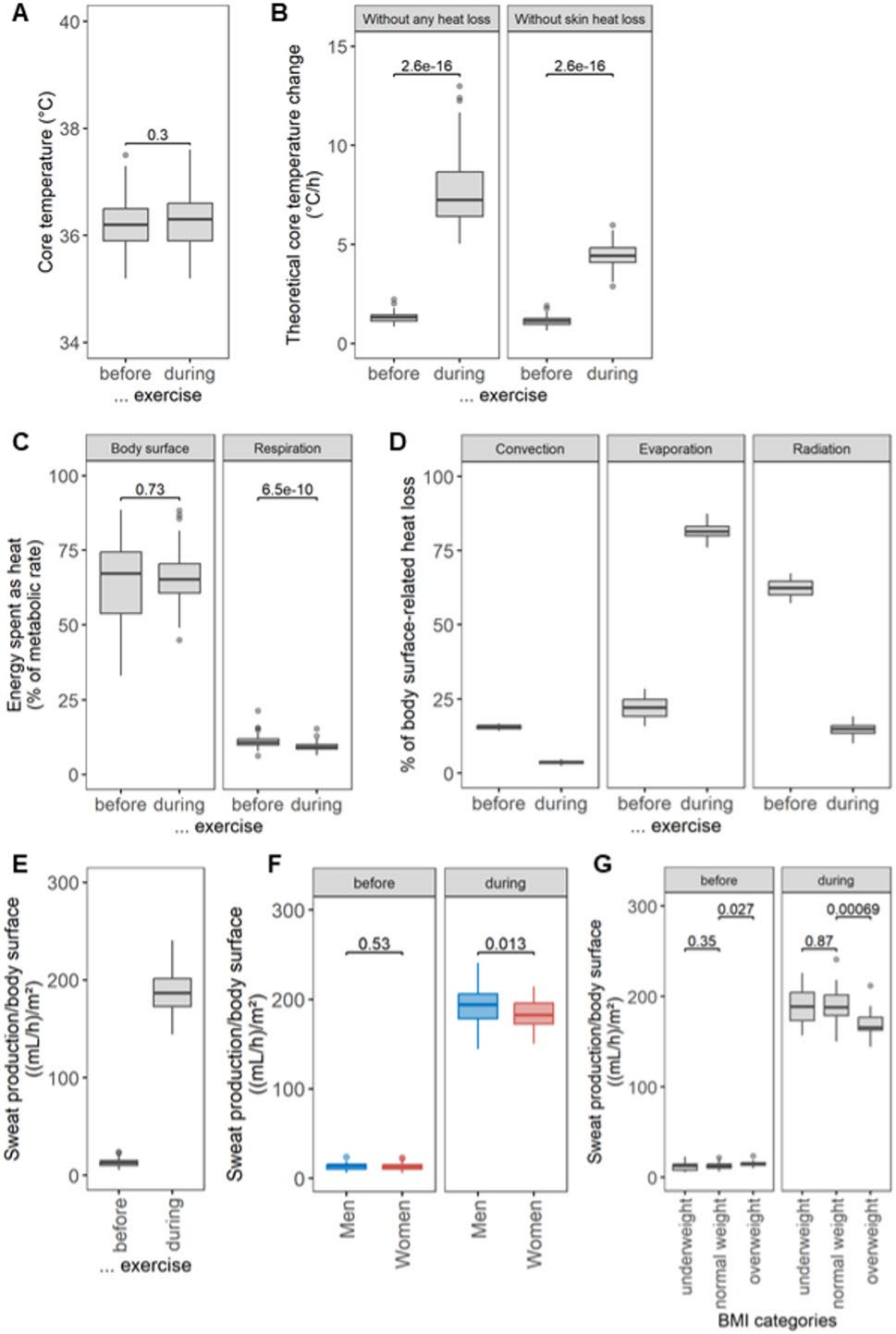
Exercise-induced changes in body temperature and heat dissipation mechanisms. **A** Core temperature during the resting and exercise phases. **B** Theoretical core temperature increase if the heat loss mechanisms of the body were impaired. **C** The relative amount of heat dissipated through the skin and by breathing. **D** The way heat is dissipated through the body surface at rest and during exercise. **E** Estimated sweat production. **F** Estimated sweat production by men and women. **G** Sweat production by individuals in different BMI categories, before and during exercise. (Number of participants *N* = 89)

The stability of the core temperature during the exercise phase shown for one practical class is confirmed when taking into account the findings of the large cohort (Figs. 2C and 5A). However, we calculate the theoretical core temperature changes that would occur if the body’s heat loss mechanisms were impaired, to demonstrate to students the absolute necessity of an efficient thermoregulation (Fig. 5B). We distinguish two scenarios: without any heat loss mechanisms (can theoretically occur in environments with high temperatures but also high ambient humidity) and without skin heat loss only (can occur in environments with moderate ambient humidity but extreme ambient temperatures combined with e.g. inappropriate clothing, or in case of patients with e.g. impaired vasodilation/vasoconstriction or reduced sweating capacity). The dramatic increase that would be caused by moderate-exercise is often underestimated by the students. But the fact that the core temperature would increase of more than 1 °C/h even at rest comes even more as a surprise.

The data collected by the students also clearly show them that the body mostly spent energy as heat, no matter if they move or not (Fig. 5C). However, the way through which the heat is dissipated clearly changes between both states (Fig. 5D), and the drastic overtake of the heat loss by evaporation is obviously correlated with an increase in sweat production (Fig. 5E). The sex-differentiating data show that women sweat slightly less during exercise than men, meaning that their cooling mechanisms are not as efficient as that of men (Fig. 5F). Finally, we observe that the persons in the overweight group produce more sweat at rest, what could be explained by an already-documented higher sympathetic activity in such persons [23] (Fig. 5G). However, during exercise, the situation is reversed. This is explained by the fact that their relative exercise-induced increase in metabolic rate (Δmetabolic rate / power, Fig. 4G) does not differ from the other BMI categories, while the body surface area through which they can dissipate heat and produce sweat is larger (Fig. 4H).

The discussion based on the exercise-induced changes thus rises additional important notions.

- Even a moderate-intensity physical activity is already associated with a strong adjustment in energy turnover (Fig. 4).
- Even during physical activity, most of the bodily energy is spent as heat and only about 25% is actually invested in muscle work (Fig. 4F).
- A failing thermoregulation quickly turns out to be dramatic for the body, even at rest (Fig. 5B).

## Discussion

Emerging global challenges, including frequent heat waves, aging and/or overweight populations, require medical staff with a deep understanding of the physiological regulation of energy metabolism and thermoregulation. Therefore, we have designed a practical course to provide students with self-experienced knowledge of the strategies the body has to cope with such challenging situations.

We planned it as a practical class in order to move from passive learning (such as listening to the corresponding lectures) to actual active participation. Studies have highlighted the positive impact of learning-by-doing over learning-by-watching or listening on performance and on transfer of knowledge from educational curriculum to professional practice [11, 12]. We therefore aimed to engage the students on different levels, including data generation, analysis and interpretation, assuming that taking part in these aspects and in-group reasoning would have a positive impact on their understanding of the tested concepts. All the variables calculated and analyzed by the students are summarized in Table 4.

**Table 4:** Summary of the calculated variables

Variable	Equation	Units	Key assumptions
Body surface	body weight^0.425^ [kg] × body height^0.725^ [cm] × 0.007184	m²	Depends on body weight and body height
Metabolic rate	O_2_-uptake [L/min] × caloric equivalent [kJ/L(O_2_)] × 1000/60	J/S	Strictly associated to the oxygen uptake
Radiative heat loss rate	radiative heat-transfer coefficient [J/(s × °C × m²)] × temperature difference [°C] × body surface available for radiative heat exchange [m²]	J/S	• Depends on the body surface area involved in heat dispersion and on the air temperature • Corresponds to ≈ 65% and ≈ 10% of the total body surface-associated heat loss at rest and during exercise, respectively
Convective heat loss rate	convective heat-transfer coefficient [J/(s × °C × m²)] × temperature difference [°C] × body surface available for convective heat exchange [m²]	J/S	• Depends on the body surface area involved in heat dispersion and on the air temperature • Corresponds to ≈ 15% and ≈ 10% of the total body surface-associated heat loss at rest and during exercise, respectively
Evaporative heat loss rate	evaporative heat-transfer coefficient [J/(s × °C × m²)] × H_2_O-vapor pressure difference [mm Hg] × body surface available for evaporative heat exchange [m²]	J/S	• Depends on the body surface area involved in heat dispersion and on the ambient humidity • Corresponds to ≈ 20% and ≈ 80% of the total body surface-associated heat loss at rest and during exercise, respectively
Total body surface-associated heat loss	radiative heat loss rate + convective heat loss rate + evaporative heat loss rate	J/S	• Depends on the amount of energy lost through the various heat dispersion pathways associated with the skin • Corresponds to ≈ 70–80% of the metabolic rate (at rest or during exercise)
Respiratory heat loss	(Minute ventilation [L/min] / 60) × (H_2_O-vapor in exhaled air - H_2_O-vapor in inhaled air [mg(H_2_O)/L(air)]) × heat of water vaporization [kJ/g(H_2_O)]	J/S	• Depends on the respiratory rate • Corresponds to ≈ 10–15% of the metabolic rate (at rest or during exercise)
Non-heat or breathing-related metabolic rate	Metabolic rate [J/s] - total body surface-associated heat loss [J/s] - respiratory heat loss [J/s] - power in ergometer [J/s]	J/S	Estimate of the fraction of metabolic rate related to basic bodily functions rather than to heat loss or muscle work
Net efficiency	100 × power in ergometer [J/s] / (MR_during_ [J/s] – MR_before_ [J/s])	%	As per definition
Theoretical core temperature increase	3.6 x total heat loss [J/s] / (body heat capacity [kJ/(kg x °C)] x body weight [kg])	°C/h	Depends on the amount of energy required to increase the temperature of 1 kg of body mass by 1 °C
Sweat production rate	Evaporative heat loss rate [J/s] x 3600 / heat of evaporation of sweat [J/mL]	mL/h	Depends on the amount of energy needed for 1 mL of water to evaporation

This course is well received and appreciated by students, according to a web-based evaluation performed at the end of the semesters (Table 5). Moreover, 99% of the students successfully pass the quiz at the end of the class, showing a good understanding of the topics covered during the day (example test provided in Supplementary Material).

**Table 5:** Evaluation of the course. The students can willingly participate to an evaluation at the end of the semester. This evaluation is web-based and the participants choose ratings between 1 (very good) and 5 (very poor). These results were obtained for the summer semester of 2025. (Total number of students enrolled in human medicine studies that semester *N* = 219)

	Rating (corresponding assessment)					Number of voters (participation rate)	
	1 (very good)	2	3	4	5 (very poor)		
Overall assessment of the course	49%	43%	8%	0%	0%	82 (37%)	
Learning effect of the course	53%	33%	11%	3%	0%	66 (30%)	

A lot of data are generated during one single class and the overall data set keeps getting larger from class-to-class. It is the task of the students and their teacher to extract the key messages from it. We therefore recommend to avoid a frontal approach for the discussion but rather recommend an interactive work during the discussion of the results. This benefits the engagement, the understanding and the memory process of the students. Nevertheless, the instructor needs to pro-actively lead the discussion and decide on which points to focus with their students of the day. Indeed, covering all aspects mentioned here can overwhelm the students and therefore prevent any additional learning effect. The teacher’s choice depends in large parts on the points that were misunderstood and need clarification, but also on students’ performance and interest. This dynamic aspect of the class can turn out to be a challenge for (beginner) instructors, since it requires spontaneity and a good knowledge of the topic. However, this also gives them more flexibility and the opportunity to provide personalized teaching suited to the audience in front of them.

Once the teacher has picked a focus topic, the relevant data is presented to the students. The teacher then takes on the role of facilitator whilst the students interpret and discuss the data in small groups, pooling their knowledge and ideas (in line with the principle of ‘problem-based learning’). Each group’s conclusions are then discussed with the whole class in order to compare them, exchange different ideas, and take the discussion further.

Using a rather easy-to-setup experiment, we can demonstrate to the students which factors influence the metabolic rate, including the body size and eventual overweight. As emphasized in the introduction, the proportion of overweight or even obese individuals among the patients of these future doctors will be significant. It is therefore crucial for them to understand what this overweight means for the body homeostasis, in order to offer their patients appropriate care and treatments, and finally collectively participate in rectifying the weight- and obesity-associated stigma [24].

In addition to the influence of different body types, we also use this data set to raise awareness among the students of sex-related differences in physiological processes. Indeed, despite a still existing sex data gap, it has been established that men and women have different prevalence rates, can develop different symptoms for a given disease and respond differently to the drugs prescribed [25]. This is especially true when considering metabolic disorders such as obesity or the associated type 2 diabetes, since women are more susceptible and have a greater risk of developing or even succumbing to the associated cardiovascular risks [26–28]. There is therefore an immediate need to apply medicine that is sensitive to sex and gender, and thus a need to train future doctors accordingly. With their own dataset, medical students can see for instance that the women-associated lower metabolic rate at rest is actually correlated with a small body surface area and therefore less heat loss. In addition, the role of differences in body tissue composition can be discussed during the class.

Finally, a beneficial “side-effect” of letting students gather data mostly in an autonomous manner is the opportunity to discuss the difficulty and challenges that come with the acquisition of reliable and valid data. A point that is especially discussed is the difference between individuals and the variation between measurements on one single person (inter- and intra-individual differences). For instance, the core temperature measurement with an infrared thermometer should theoretically not be an issue, but the variation of the values perfectly illustrates that the need to reproduce some measurements several times (technical replicates) and to perform them with a sufficient number of test subjects (biological replicates and power test). These aspects go way beyond discussing if results gathered during a university semester are statistically significant or not, but it is a chance to raise awareness among future medical staff of critical criteria to determine if a dataset is reliable. Considering that statistically illiteracy of clinicians has been documented and associated with consequences for their patients’ health [29, 30], we consider this aspect of our discussion with students to be a clear benefit.

### Limitations and future considerations

Our approach and the proposed calculations regarding heat expenditure can only be applied if the experiments take place in an environment allowing heat dissipation through radiation, convection and sweating (i.e. without a saturated humidity), but without fostering one specific process. For instance, working in a strongly air-conditioned room, where students are below their thermal comfort zone at rest, may prevent the direct translations of our approach as dry-heat exchange mechanisms (radiation and convection) may then prevail over evaporation [31].

Although the autonomous work and data gathering by the students give us the opportunity to discuss data variability and sample size as discussed hereinabove, the quality of the data can still remain an issue sometimes. Indeed, despite regular protocol revisions to clarify potentially confusing points, the teachers still need to check the data collected by the students before moving along with discussion and integration in the larger datasets.

The data acquired during the classes and the subsequent discussions may be typical for a rather homogeneous, young, and mostly healthy cohort. We are nonetheless aware that some aspects cannot be illustrated to the students with their own work, particularly the influence of ageing. Additionally, few test persons were actually overweight and even less obese, although the prevalence of adults with a BMI above 25 exceeds 20% in Germany [1]. Reports showing that German medical students are not representative of the general population actually exist, particularly as most students come from a privileged socio-economic background [32, 33], which has been correlated with a lower prevalence of obesity in developed countries [34]. As mentioned, it is vital for future practitioners to understand the impact of body type on physiological aspects, and the data we collected here open the doors for such discussions, but there is still room to raise even more awareness. This could be achieved by collecting additional data outside of the practical class context with a more diverse population, but the didactic aspect might get lost. Another aspect that could be considered is to test similar or close experiment set-ups while doing an obesity simulation (with e.g. the test person wearing a fat suit or additional weight).

## Conclusions

Here we describe an easy-to-implement practical course that illustrates the importance of the currently relevant physiological topics that are metabolic balance and thermoregulation. One of the strengths of this course is that it uses data generated by students to facilitate in-depth discussions and teach them about complex, systemic regulatory mechanisms. This course has received very positive evaluations from the medical students in recent years.

## Supplementary Information

Supplementary Material 1.

Supplementary Material 2.

Supplementary Material 3.

Supplementary Material 4.

Supplementary Material 5.

## Data Availability

The metadata used to generate the shown figures (contains the raw and processed data) and the data sheet provided to the students have been made publicly available (Figshare data: 10.6084/m9.figshare.31398534).
