# Supplementary material for: A practical approach to illustrate the importance of the bodily energy and heat balances and of the associated regulatory loops to healthcare students
Source: BMC Med Educ. 2026 Jul 20;26:1186. doi: 10.1186/s12909-026-09954-6 (PMC13386703; doi:10.1186/s12909-026-09954-6)
Supplement: Supplementary file 1 — Supplementary Material 1. [file 12909_2026_9954_MOESM1_ESM.pdf]

Name	

	before exercise (4 min)	during exercise (12 min)
**minute ventilation VE [l/min]**		
**O_2-uptake VO_2 [lO_2/min]**		
**heart rate HF [1/min]**		
**skin temperature [°C]**		
**core temperature [°C]**		
**cycle power [W]**		
**oxygen debt[l]**		
**clearance of oxygen dept [l]**		

Tab. 1: Measured values and results concerning O_2-balance (from program "Metasoft").

**room temperature**	**[°C]**		
**humidity**	**[%]**		
**body height**	**[cm]**		
**body mass**	**[kg]**		
**body surface area BSA**<br>$BSA = body\ mass\ [kg]^{0{,}425} \cdot height\ [cm]^{0{,}725} \cdot 0{,}007184$	**[m^2]**		
**body surface available for radiative heat exchange** *(0,5 · BSA)*	**[m^2]**		
**body surface available for convective heat exchange** *(0,3 · BSA)*	**[m^2]**		
**body surface available for evaporative heat exchange**	**[m^2]**	**before exercise** *(0,3 · BSA)*	**during exercise** *(0,9 · BSA)*

Tab. 2: Data collection for further calculations.

	before exercise	during exercise
**metabolic rate MR [J/s]**		
MR [J/s] = (O_2-uptake [l/min] · caloric equivalent [kJ/lO_2] · 1000)/60 (caloric equivalent ≈ 20 kJ/lO_2)		
**net efficiency [%]**		
net efficiency [%] = 100 · cycle power [W] / MR during exercise [J/s]		

Tab. 3: Calculation of the metabolic rates and net efficiency.

Calculate in table 4, how much heat is lost through radiation, convection and evaporation. Use the respective body surface available for heat exchange from table 3.

The differences in temperature / H_2O-vapor pressure are to be gathered between skin and air. See provided detailed formulas for heat loss.

		before exercise	during exercise
**radiative heat loss rate**	**[J/s]**		
radiative heat loss rate [J/s] = 6 [J/(m² · s · K)] · difference in temperature [K] · body surface available [m²]			
**convective heat loss rate**	**[J/s]**		
convective heat loss rate [J/s] = 2,5 [J/(m² · s · K)] · difference in temperature [K] · body surface available [m²]			
**evaporative heat loss rate**	**[J/s]**		
evaporative heat loss rate [J/s] = 40 [J/(m² · s · kPa)] · difference in H_2O-vapor pressure [kPa] · body surface available [m²]			

saturation vapor pressure E_S of H_2O-vapor at different temperatures T:

T [℃]	18	19	20	21	22	23	24	25	26	27	28	29	30	31	32	33	34	35	36	37
E_S [kPa]	2,06	2,20	2,34	2,49	2,64	2,81	2,98	3,17	3,34	3,54	3,76	3,98	4,22	4,49	4,75	5,03	5,32	5,62	5,94	6,27

actual H_2O-vapor pressure = saturation vapor pressure · relative humidity.

Please use the humidity as stated from the hygrometer. Before exercise use the same value for skin and air. During exercise the relative humidity of the skin is presumed 100 %.

		before exercise	during exercise
**heat retention during exercise**	**[J/s]**		
heat retention during exercise [J/s] = 3500 [J/kg · K] · body mass [kg] · (core temperature$_{exercise}$ – core temperature$_{rest}$ [°C]) / 480 s			
**Total body surface-associated heat loss**	**[J/s]**		
**amount radiation (of heat loss)**	**[%]**		
**amount convection (of heat loss)**	**[%]**		
**amount evaporation (of heat loss)**	**[%]**		
**amount of energy spent as heat**	**[%]**		

Tab. 4: Calculation of body surface-associated heat loss
