# Supplementary material for: A practical approach to illustrate the importance of the bodily energy and heat balances and of the associated regulatory loops to healthcare students
Source: BMC Med Educ. 2026 Jul 20;26:1186. doi: 10.1186/s12909-026-09954-6 (PMC13386703; doi:10.1186/s12909-026-09954-6)
Supplement: Supplementary file 2 — Supplementary Material 2. [file 12909_2026_9954_MOESM2_ESM.pdf]

	test subject 1		test subject 2		test subject 3		test subject 4	
Name								
Sex (m/w)								
	before	during	before	during	before	during	before	during
Minute ventilation (L/min)								
Oxygen uptake (L/min)								
Heart rate (1/min)								
Skin temperature (°C)								
Core temperature (°C)								
Work in ergometer (W)								
Oxygen deficit (L)								
Excess post-exercise oxygen consumption (L)								
Room temperature (°C)								
Humidity of ambient air (%)								
Body height (m)								
Body mass (kg)								
Body surface (m^2)	0,00		0,00		0,00		0,00	
Effective surface area (radiation) (m^2)	0,00		0,00		0,00		0,00	
Effective surface area (convection) (m^2)	0,00		0,00		0,00		0,00	
Effective surface area (evaporation) (m^2)	0,00	0,00	0,00	0,00	0,00	0,00	0,00	0,00
Metabolic rate (J/s)	0	0	0	0	0	0	0	0
Energy conversion efficiency (%)		#DIV/0!		#DIV/0!		#DIV/0!		#DIV/0!
Heat loss by radiation (J/s)	0	0	0	0	0	0	0	0
Heat loss by convection (J/s)	0,0	0	0,0	0	0,0	0	0,0	0
Heat loss by evaporation (J/s)	0	0	0	0	0	0	0	0
Accumulation of heat due to exercise (J/s)		0		0		0		0
Total heat loss (J/s)	0	0	0	0	0	0	0	0
Relative radiative heat loss (%)	#DIV/0!	#DIV/0!	#DIV/0!	#DIV/0!	#DIV/0!	#DIV/0!	#DIV/0!	#DIV/0!
Relative convective heat loss (%)	#DIV/0!	#DIV/0!	#DIV/0!	#DIV/0!	#DIV/0!	#DIV/0!	#DIV/0!	#DIV/0!
Relative evaporative heat loss (%)	#DIV/0!	#DIV/0!	#DIV/0!	#DIV/0!	#DIV/0!	#DIV/0!	#DIV/0!	#DIV/0!
Relative fraction of metabolic rate spent as heat (%)	#DIV/0!	#DIV/0!	#DIV/0!	#DIV/0!	#DIV/0!	#DIV/0!	#DIV/0!	#DIV/0!
Potential change of core temperature in case of inhibited heat loss (°C/h)	#DIV/0!	#DIV/0!	#DIV/0!	#DIV/0!	#DIV/0!	#DIV/0!	#DIV/0!	#DIV/0!
Sweat production (mL/h)	0	0	0	0	0	0	0	0
BMI (kg/m^2)	#DIV/0!		#DIV/0!		#DIV/0!		#DIV/0!	
Oxygen intake (ml/(min · kgBW))	#DIV/0!	#DIV/0!	#DIV/0!	#DIV/0!	#DIV/0!	#DIV/0!	#DIV/0!	#DIV/0!
Saturation vapor pressure in air (KPa)	0,61		0,61		0,61		0,61	
Actual vapor pressure in air (KPa)	0,00		0,00		0,00		0,00	
Saturation vapor pressure on skin (KPa)	0,61	0,61	0,61	0,61	0,61	0,61	0,61	0,61
Actual vapor pressure on skin (KPa)	0,00	0,61	0,00	0,61	0,00	0,61	0,00	0,61
