# Supplementary material for: A practical approach to illustrate the importance of the bodily energy and heat balances and of the associated regulatory loops to healthcare students
Source: BMC Med Educ. 2026 Jul 20;26:1186. doi: 10.1186/s12909-026-09954-6 (PMC13386703; doi:10.1186/s12909-026-09954-6)
Supplement: Supplementary file 3 — Supplementary Material 3. [file 12909_2026_9954_MOESM3_ESM.pdf]

Consideration of the work of breathing

A perfect comprehensive approach would consider the energy spent by the work of breathing. The latter is however difficult to estimate, and when it is done, it turns out to have a very limited contribution to the energy expenditure (calculated here as below 1% of the resting metabolic rate). We thus leave it out of the manuscript and of the described approach. We propose here nonetheless a way to estimate it the readers are interested in implementing it.

The work of breathing is calculated based on variations of tidal volumes (ΔV) and pressures (ΔP, intrapulmonary or pleural) described in textbooks.

Work of breathing [J/s] = static work of breath [J/s] + dynamic work of breath [J/s]

= (ΔV [L] × ΔP_{pleura} [kPa] x respiratory rate [breath per minute]) + (ΔV [L] × $\Delta P_{intrapulmonary}$ [kPa] x respiratory rate [breath per minute])

with ΔV = 1 L before and 2 L during exercise, $\Delta P_{intrapulmonary}$ = 0.2 before and 0.4 during exercise, ΔP_{pleura} = 0.8 before and 1.6 during exercise, and respiratory rate = 10 before and 20 during exercise.
