# Supplementary material for: A practical approach to illustrate the importance of the bodily energy and heat balances and of the associated regulatory loops to healthcare students
Source: BMC Med Educ. 2026 Jul 20;26:1186. doi: 10.1186/s12909-026-09954-6 (PMC13386703; doi:10.1186/s12909-026-09954-6)
Supplement: Supplementary file 4 — Supplementary Material 4. [file 12909_2026_9954_MOESM4_ESM.pdf]

Example for an evaluation sheet with 4 multiple-choice questions

1. Which statement about heat production or heat loss of the adult human body is most accurate?

(A) When the ambient temperature increases from 37 °C to 80 °C, heat loss by radiation increases linearly.

(B) At low air humidity, heat loss through sweating is possible even at an ambient temperature above 37 °C.

(C) At low air humidity and 37°C, most of the body heat is lost through breathing.

(D) Heat production due to shivering generally reaches 25 times the heat production of the basal metabolic rate.

(E) Heat production in brown adipose tissue generally reaches 10 times the heat production of the basal metabolic rate.

2. The energy expenditure of a person is to be determined using indirect calorimetry. Which of the following parameters is required for it?

(A) EMG (electromyogram) of the leg muscles

(B) Total O_2 consumption

(C) Concentration of free thyroxine (fT4) in blood plasma

(D) Core body temperature

(E) Vital capacity

3. For a test person during a practical course, the following values were determined:

respiratory quotient RQ = 0.9

CO_2 output = 1.2 L/min

caloric equivalent = 20 kJ/L O_2.

How high is the energy expenditure?

(A) 167 W

(B) 334 W

(C) 85 W

(D) 667 W

(E) 444 W

4. Under which of the following environmental conditions is prolonged physical exercise (such as running a marathon) most severely restricted?

(A) No wind, 20°C, 15% relative humidity

(B) 33°C, wind speed 5 km/h and 95% relative humidity

(C) Wind speed 15 km/h, 25% relative humidity and 15°C

(D) 32°C, 5% relative humidity and wind speed 40 km/h

(E) Wind speed 20 km/h, 40% relative humidity and 25°C
